# Resin Nanoceramic CAD/CAM Restoration of the Primary Molar: 3-Year Follow-Up Study

**DOI:** 10.1155/2017/3517187

**Published:** 2017-06-20

**Authors:** Akif Demirel, Tuğba Bezgin, Funda Akaltan, Şaziye Sarı

**Affiliations:** ^1^Department of Pediatric Dentistry, Faculty of Dentistry, Ankara University, Ankara, Turkey; ^2^Department of Prosthodontics, Faculty of Dentistry, Ankara University, Ankara, Turkey

## Abstract

This case report presents the clinical use of a resin nanoceramic CAD/CAM restoration of a primary second molar without successor in the form of a permanent second premolar tooth in a patient. Three-year follow-up of the case revealed that resin nanoceramic CAD/CAM restoration of the primary molar without successor achieved both aesthetics and function. Despite the high cost of treatment, this type of restoration should be considered if the retained tooth is expected to maintain functionality over the long term.

## 1. Introduction

Third molar teeth aside, mandibular second premolars are the most common congenitally absent teeth [1–3]. In such cases, treatment planning is affected by a variety of factors, including patient age, developmental levels of adjacent teeth, facial profile, arch length, incisor inclination, jaw protrusion, degree of infraocclusion in persistent deciduous teeth, and root resorption [1, 3].

When a persistent deciduous tooth is extracted at an early stage (ages 8-9), the resultant space can undergo spontaneous closure through mesial movement of adjacent teeth. However, this can only occur if the first permanent molar root-formation is incomplete [4–6]. Moreover, extraction is not recommended in patients with generalized spacing of teeth, deep-bite, hyperdivergent facial profile, or mandibular retrusion because it will damage the facial profile [7]. In older patients, the extraction space can be closed orthodontically [1, 8] or restored with a prosthetic tooth implant or autotransplantation after the completion of growth [1, 5, 9, 10].

If there is no specific indication for extraction, another treatment alternative is to leave the persistent deciduous tooth in place, if the tooth has good (or at least acceptable) crown-root structure, functionality, and aesthetics. This treatment preserves the integrity of the alveolar bone for a future implant procedure [11]. However, because the space resulting from the extraction of a primary molar tooth tends to be too wide for a premolar implant as well as too narrow for a molar implant [8, 11, 12], reducing the mesiodistal width of the retained deciduous molar to that of a second permanent premolar and allowing for spontaneous closure of the remaining minor space through mesial movement of adjacent teeth are recommended [8, 12].

Because mesiodistal reduction of the deciduous tooth leaves it with exposed dentin that is prone to dental caries, composite restoration of the exposed dentin surface is recommended as a preventative measure [8]. However, composite restoration entails the risk of marginal leakage and colorization over the long term [13]. Computer-aided design or computer-aided manufacturing (CAD/CAM) offers a restoration method that decreases the risk of human error and provides highly aesthetic outcomes, albeit with relatively high costs [14]. Initially, ceramic blocks were used [15, 16], but today they have been largely replaced by composite blocks [16–18] that are easier to process and repair and cause much less abrasion of teeth in the opposing arch [18–20]. While CAD/CAM restoration has become a common method of treatment for permanent teeth in children, there are only a limited numbers of case reports on its use in deciduous teeth [21].

The case report below presents the clinical use of a resin nanoceramic CAD/CAM restoration of a primary second premolar without successor in the form of a permanent second premolar tooth in a patient.

## 2. Case Report

**Figure 1:**
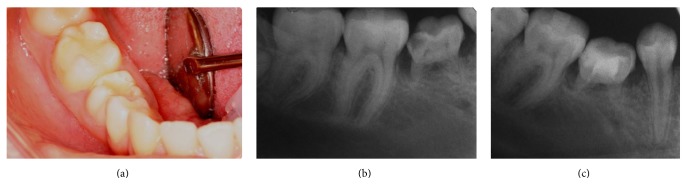
Preoperative intraoral and radiographic views of the patient. (a) Preoperative intraoral view. (b) Preoperative periapical radiograph. (c) Postoperative radiograph after root-canal treatment.

A 13-year-old female patient presented at the Department of Pedodontics, Faculty of Dentistry, Ankara University, complaining of the delated eruption of a permanent tooth. The patient's medical history indicated that one month earlier she had received antibiotics to treat an abscess of the right mandibular primary second molar tooth. Clinical examination showed deep dentinal caries and a 1 mm infraocclusion when compared to the adjacent teeth (Figure 1(a)). Radiographic examination showed no permanent tooth germ under the primary second molar, no periradicular lesion, and uniform bone between the primary second molar tooth and the first permanent premolar (Figures 1(b) and 1(c)). Both clinical and radiographic examinations showed no ankylosis. Additionally, on the other quadrant, congenital agenesis of lower second premolar was observed and primary second molar was previously extracted (Figure 3(a)). As a result of this extraction, mesialization of permanent first molar was seen and residual space was in size of second premolar mesiodistal dimension. The patient had a Class I molar relationship. Given the patient's age, a treatment plan that included extraction of right primary second molar followed by orthodontic treatment to close the extraction gaps was recommended; however, this plan was rejected by the patient. An alternative treatment option described below was presented to the parents, and after approval written consent was obtained for treatment.

Pulpectomy was performed as described below. Inferior alveolar nerve block was administered (2% lidocaine with 1 : 100.000 adrenaline), the tooth was isolated with a rubber dam, the pulp chamber was accessed, and the working length was determined using a Size 15 sterile K-file to 2 mm short of the radiographic apex. Intracanal tissue was extirpated using a barbed broach (Medin Barbed Broach, Vlachovice, Czech Republic), and the canals were filed with K-Flexofiles until a master file size of 30 was reached (G-star Medical Co., Ltd., Guangdong, China). Canals were irrigated with 2 ml of 1% sodium hypochlorite (NaOCl) between instruments and with 5 ml of sterile saline as a final irrigation. Canals were dried with premeasured paper points up to 2 mm from the root apices. Canals were filled with white mineral trioxide aggregate (WMTA) (ProRoot, Dentsply, Tulsa Dental, OK, USA) prepared according to the manufacturer's instructions using a lentulo and hand tools. After radiographic control of the root-canal filling, a wet cotton pellet was applied to the pulp chamber, and the access cavity was sealed with glass ionomer cement (Ionofil Plus, Voco, Cuxhaven, Germany). Two days later, the cotton pellet was removed, and the cavity was restored with glass ionomer cement and compomer (Dyract XP, Dentsply, Tulsa Dental, OK, USA).

**Figure 2:**
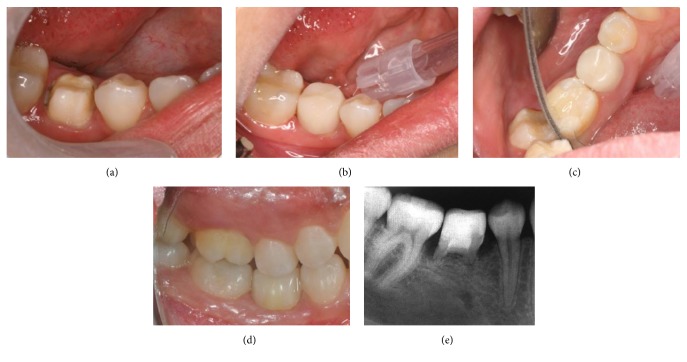
Crown preparation and cementation. (a) Tooth preparation with chamfered margin. (b–d) Intraoral views of the crown after cementation. (e) Postoperative radiograph of the crown.

**Figure 3:**
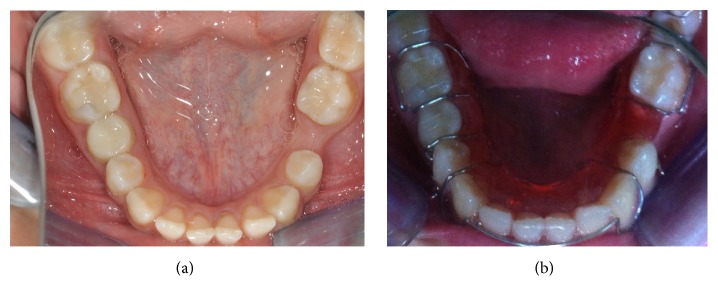
Intraoral view of left primary second molar extraction space (a). Removable space maintainer for space maintenance (b).

In order to accommodate any future dental implant that might be required in the event of the loss of the retained primary molar, it is planned to restore the tooth RNC CAD/CAM by reducing mesiodistal width in the form of a permanent premolar. The deciduous tooth was prepared according to standardized preparation techniques with a chamfered margin. The size of the reduction was determined according to the mesiodistal and buccolingual measurements of the erupted opposite first permanent premolar tooth (mesiodistal: 8 mm; buccolingual: 8 mm). Measurements were obtained using a digital camera (Cerec AC, Bluecam, Germany), and the crown was formed from a nanoresin ceramic block (3M Lava Ultimate, United States) with milling method (Cerec MC XL Premium, Germany). The finished crown was cemented using water-based adhesive cement (Adhesor Carbofine, Spofa Dental, Czech Republic) (Figure 2). Finally, a removable space maintainer was applied for left edentulous space (Figure 3(b)).

**Figure 4:**
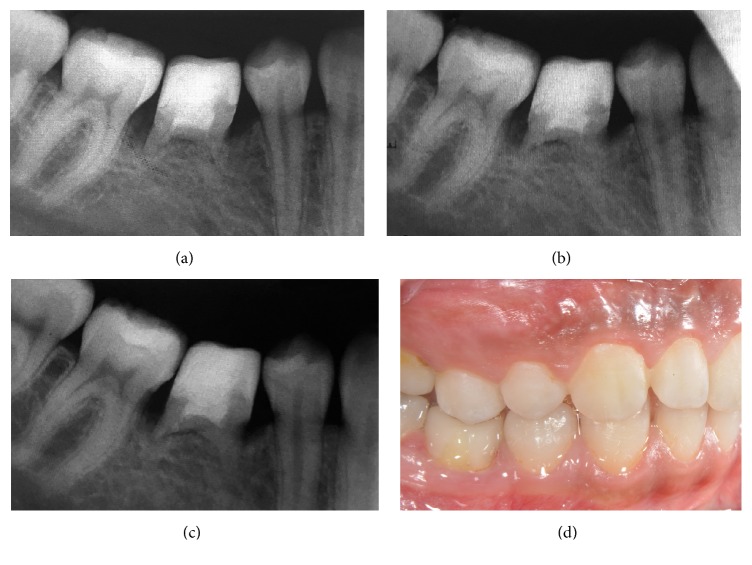
Follow-up views: (a) 1-year follow-up radiograph; (b) 2-year follow-up radiograph; (c) 3-year follow-up radiograph; (d) 3-year follow-up intraoral view.

Regular clinical and radiographic follow-ups were conducted every six months for three years (Figure 4). Clinical examination included evaluation of sensitivity to percussion and palpation, soft-tissue pathology, infraocclusion and marginal fitness, and integrity of the crown restoration. Radiographic examination included evaluation of internal and external root resorption, periradicular lesions, and ankylosis. No clinical or radiographic pathology was observed at any time during the follow-up period.

Three-year follow-up of the case revealed that resin nanoceramic CAD/CAM restoration of the primary molar without successor achieved both aesthetics and function.

## 3. Discussion

While primary second molar teeth without successors are known to remain serviceable for many years [10], if extraction is required in the future, a dental implant may be indicated [22, 23]. Successful implant treatment requires an adequate amount of bone volume mesiodistally as well as buccolingually [24]. Without adequate bone, a graft may be required, which involves additional financial costs as well as treatment time [8]. A retained primary tooth offers the advantage of helping to preserve bone and soft-tissue structure [11]; however, in view of the possible need for future implant treatment, reshaping the retained tooth to resemble a permanent premolar is recommended in order to reduce the mesiodistal width [8, 11]. In the case presented here, the decision was made to restore the second primary molar in order to avoid supraeruption of the maxillary teeth, achieve normal occlusion, and restore function and aesthetics.

In the case presented here, the decision was made to perform root-canal treatment for the second primary molar tooth because of the abscess formation history. In general, a resorbable paste or a combination of pastes such as zinc oxide eugenol (ZOE), iodoform, and calcium hydroxide is used for primary tooth pulpectomy [25]; however, in the case of a primary tooth without a successor, a nonresorbable material is recommended [26, 27]. Previous studies have reported success with a combination of gutta-percha and ZOE sealer [28] and with mineral trioxide aggregate (MTA) [26, 29]. In this case, MTA was selected as a root-filling material because of its biocompatibility, excellent sealing ability, and long-term better prognosis than gutta-percha and ZOE sealer [27].

Dental composites, stainless steel crowns, and gold onlays can all be used to restore and reshape second primary molars without successors [11, 30, 31]. However, composite resin restorations still have the drawbacks of marginal leakage and color changes with foods and beverages, whereas stainless steel crowns offer poor aesthetics [13, 32, 33]. In the case presented here, due to the continuing growth of the patient, the restoration was expected to remain in service for approximately 5 years until implant placement; therefore, a resin CAD/CAM restoration was performed.

Unlike composite restorations, resin nanoceramic blocks offer optimized mechanical properties with a higher degree of monomer polymerization and less abrasion of the opposing dental arch when compared to ceramic restorations; they are also easily repaired using composite resin, if necessary [18–20]. While the aesthetic advantages of full ceramic restorations are well known [18], resin nanoceramic (Lava Ultimate) restorations also have excellent aesthetic results [34]. For this reason, a CAD/CAM Cerec composite block was used in the present case to reshape a primary second molar to mimic the morphology of a permanent second premolar.

Three-year follow-up of the case revealed that resin nanoceramic CAD/CAM restoration of the primary molar without successor achieved both aesthetics and function. However, longer follow-up is needed to evaluate for a possible ankylosis formation in the future. The patient's occlusion will be suitable for an implant restoration if needed in the future.

## 4. Conclusion

As this case report clearly shows, agenesis of a permanent mandibular second premolar can be treated by reshaping a retained second primary molar using a resin nanoceramic CAD/CAM restoration to achieve good function and esthetics. Despite the high cost of treatment, this alternative should be considered if the retained tooth is expected to maintain functionality over the long term. Despite the high cost of treatment, this type of restoration should be considered if the retained tooth is expected to maintain functionality over the long term.
